# Understanding mistreatment during institutional delivery in Northeast Nigeria: a mixed-method study

**DOI:** 10.1186/s12978-019-0837-z

**Published:** 2019-12-02

**Authors:** Nasir Umar, Deepthi Wickremasinghe, Zelee Hill, Umar Adamu Usman, Tanya Marchant

**Affiliations:** 1 Department of Disease Control, London School of Hygiene & Tropical Medicine, London, WC1E 7HT UK; 2 Institute for Global Health, University College London, London, UK; 3 Data Research & Mapping Consult, Abuja, Nigeria

**Keywords:** Quality of care, Respectful maternity care, Mistreatment, Maternal and newborn health, Mixed-methods

## Abstract

**Background:**

Improving quality of care including the clinical aspects and the experience of care has been advocated for improved coverage and better childbirth outcomes.

**Objective:**

This study aimed to explore the quality of care relating to the prevalence and manifestations of mistreatment during institutional birth in Gombe State, northeast Nigeria, an area of low institutional delivery coverage.

**Methods:**

The frequency of dimensions of mistreatment experienced by women delivering in 10 health facilities of Gombe State were quantitatively captured during exit interviews with 342 women in July–August 2017. Manifestations of mistreatment were qualitatively explored through in-depth interviews and focus groups with 63 women living in communities with high and low coverage of institutional deliveries.

**Results:**

The quantitative data showed that at least one dimension of mistreatment was reported by 66% (95% confidence interval (CI) 45–82%) of women exiting a health facility after delivery. Mistreatment related to health system conditions and constraints were reported in 50% (95% CI 31–70%) of deliveries. In the qualitative data women expressed frustration at being urged to deliver at the health facility only to be physically or verbally mistreated, blamed for poor birth outcomes, discriminated against because of their background, left to deliver without assistance or with inadequate support, travelling long distances to the facility only to find staff unavailable, or being charged unjustified amount of money for delivery.

**Conclusions:**

Mistreatment during institutional delivery in Gombe State is highly prevalent and predominantly relates to mistreatment arising from both health system constraints as well as health worker behaviours, limiting efforts to increase coverage of institutional delivery. To address mistreatment during institutional births, strategies that emphasise a broader health systems approach, tackle multiple causes, integrate a detailed understanding of the local context and have buy-in from grassroots-level stakeholders are recommended.

## Plain English summary

There is growing evidence from Nigeria and around the world that women who deliver their babies in health facilities can experience mistreatment. In our study, we describe how frequently this happens in Gombe state, north-eastern Nigeria, and what type of mistreatments women experience. Using a questionnaire, we interviewed consenting women as they left the facility after birth and asked them about events that occurred during their labour and delivery, and their perception of the care that health workers provided. In addition, through in-depth interviews and focus group discussions, we talked to women with young infants about mistreatment at birth and asked them to try to explain their experiences to us. At least one type of mistreatment was reported by 66% of the women. About 50% of mothers experienced mistreatment due to poor health system conditions and constraints, for example staff shortages or staff not having the commodities they needed to provide care. And 46% experienced mistreatment related to having poor rapport with the provider, for example being denied a birth companion, examined without permission or not allowed to give birth in their preferred position. Both health system constraints and poor health worker behaviours limit efforts to increase coverage of institutional delivery. Immediate and sustained attention to the quality of care as it pertains to the experience of users is needed.

## Background

Institutional delivery is advocated to improve maternal and newborn health outcomes [1, 2]. Globally, the majority of births now occur in a health facility [3], although, high coverage is not uniform [3]. Nigeria continues to have suboptimal institutional delivery coverage, being 39% in 2018 [4]. Among the reasons consistently cited by women for not delivering in a health facility were concerns relating to a perception of poor quality of services [5, 6]. A combination of the effectiveness of care given and the negative experience from services received shapes users’ perception of care, which in turn influences health-seeking behaviour [7].

Considering every pregnant woman is at risk of obstetric complication [8, 9], access to timely and appropriate obstetric care remains imperative [10, 11]. Health systems should therefore, strive to improve the quality of care provided to women during institutional delivery, both in terms of process quality and experience of care, to guarantee the fundamental rights of women, encourage utilisation and ensure improved outcomes [12–14].

One limitation to addressing the negative experience of care has been the difficulty in defining and measuring the problem [15, 16]. But, recent developments from the characterisation of disrespect and abuse by Bowser and Hill [17], and a revised typology by Bohren et al. [18], have furthered the review of quality of care as it relates to women’s experience or perception of care received during institutional delivery [12, 15, 18, 19]. These developments allow for comparison with the expected standard of care, and where the care provided falls short of agreed standards, highlights opportunities for improvement [20–23]. However, evidence from Nigeria continues to be scarce [24].

This study was conducted in Gombe state one of six northeastern states of Nigeria. The quality of maternal and newborn health (MNH) services are suboptimal in Nigeria, but also varies between states, in part due to the resources committed to health by individual states [25]. In Gombe State, more than 60% of women still deliver at home [26], in part because of cultural or religious beliefs, cost or geographical access [4, 27–30]. Infrastructural and personnel deficiencies, and attitude of health workers, have also been suggested as possible deterrents [25, 27], as might be the fear of mistreatment, although there is no evidence to support this assumption.

In this study, we aimed to explore the quality of care relating to the prevalence and manifestations of mistreatment during institutional birth in Gombe State, northeast Nigeria, where maternal mortality and morbidity is persistently high and access to health care sub-optimal [26, 31]. We describe the frequency of different dimensions of mistreatment reported by women and use qualitative methods to explore the manifestations of mistreatment in this setting.

## Methods

### Study setting

Gombe State has an estimated population of 2.6 million based on the last census in 2006. Gombe State has 11 Local Government Areas, is multi-ethnic and 80% rural [32, 33]. The state has a high maternal mortality ratio, estimated at 1549 maternal deaths per 100,000 live births, neonatal mortality is estimated at 35 neonatal deaths per 1000 live births and just 29% of women had delivered their most recent newborn in a health facility [26, 32–34]. Public health services account for 98% of institutional deliveries in the state [26, 32–34]. In Gombe State, approximately 486 public health facilities provide labour and delivery services 460 of which are primary health facilities and 28 are referral facilities offering both labour and delivery services and specialised care [35]. Lower cadre health care workers, for example, community health extension workers (CHEW), junior community health extension workers (JCHEW) and community health officers (CHO) comprise the majority of the health care workforce [36].

In primary health care facilities, nurses or midwives are responsible for the organisation and provision of MNH services with the assistance of CHEWs, JCHEWs or any lower cadre health worker available. In the absence of nurses of midwives these lower cadre health workers must take full responsibility. In most of the PHCs, health care providers are not available 24 h a day, limiting access to facility-based care in case of an emergency or a delivery outside daylight hours. This problem is less acute in referral health facilities where nurses or midwives contribute to the organisation and delivering of MNH services under the supervision of a medical doctor.

### Study design

Quantitative and qualitative data were collected in 2017, as part of a programme of work to understand the quality of maternal and newborn care in Gombe State. The quantitative study involved conducting structured exit interviews with recently delivered women upon discharge after institutional delivery, in 10 primary health care facilities. The qualitative study included in-depth interviews (IDIs) and focus groups with 63 women who had recently delivered in a health facility in two local government areas (LGAs) (districts): Kaltungo, characterised by higher levels of facility births, and Kwami, where there are low levels of facility deliveries, and are reported below in line with the Consolidated Criteria for Reporting Qualitative Research (COREQ) [37].

This research was conducted with approval from the ethics review boards of the Federal Ministry of Health Abuja, Nigeria, the State Ministry of Health Gombe State, Nigeria and the London School of Hygiene & Tropical Medicine (reference 12,181). To obtain participant’s informed consent, all potential participants were provided with study information sheet and a consent form. The information sheet provided information to the participants about their right to participate or refuse to participate in the study, the right to change their mind about participating during the course of the study, and the right to withdraw from the study at any time. The information sheet was read and explained to those participants that cannot read. The free and written informed consent of all interviewees was obtained. Participants unable to sign the consent form were allowed to thumb print, to affirm their consent.

### Quantitative data collection

A random sample of 107 health facilities was drawn from approximately 500 public primary health facilities. Volume of births occurring in the previous six months in the 107 sampled primary health facilities was determined by reviewing their maternity registers, and the 10 primary health facilities with the highest volume of births in the state were selected for the study. The 10 selected facilities had an average of 15.7 births (SD 12.0) per month, which is higher than the state-level average of 4.3 births (SD 6.3) per month in primary health facilities [35]. The facilities were primary health facilities, providing all services (e.g. primary care, pregnancy care, labour and delivery services). Emergency care and complicated cases from these health facilities are referred to referral health facilities.

All women giving birth in these facilities in July–August 2017, and who had a live newborn, were invited to complete an exit interview about events that occurred during their labour and delivery, and their perception of the care that health workers provided, including respectful care. To ensure confidentiality, the exit interviews were conducted in a separate room or area reserved for the interviews within the health facilities. In each of these 10 facilities, two trained data collectors and a supervisor were posted in shifts covering day and night deliveries, seven days a week for approximately four weeks. This was determined to be the amount of time needed in these high-volume facilities to recruit a sample of 320 births. The sample size was calculated based on the assumed prevalence of respectful maternity practices of 10–20%, a power of 80%, a 95% confidence interval.

#### Study instrument

The study instrument was a structured questionnaire covering the demographic information of study participants, the content of care provided to the mother and the newborn, and respectful care during institutional birth. The study tool was operationalised based on revised typology by Bohren et al. [18] The tool had 31 items, structured around the seven domains of mistreatment proposed by Bohren and others: physical abuse (2-items), sexual abuse (1-item), verbal abuse (2-items), stigma and discrimination (1-item), failure to meet professional standards of care (6-items), poor rapport between women and health providers (11-items), and health system conditions and constraints (8-items) [18]. Additional file 1: Table S1 present questions used to assess mistreatment during institutional delivery in Gombe State. Women were asked to provide Yes or No response to the questions listed in Additional file 1: Table S1. This method is widely used in attitude measurement, for example, in Abuya et al. [23] and easily understood by respondents [38]. The study instrument was first reviewed and validated for content in collaboration with a group of health workers (doctors, nurses and midwives) working in Gombe. The tool was pilot tested within the same study health facilities with post-partum women. The feedback from the health workers and the pilot informed further refinement and finalisation of the study instrument. Data were collected using personal digital assistants, programmed in Census and Survey Processing System (CSPro), and took about an hour to complete.

### Quantitative data analysis

Descriptive statistics about the study sample of postpartum mothers and their reported experience of respectful maternity care were tabulated. An aggregate outcome variable on the report of any abuse was computed from each of the seven domains. We used the svyset command in Stata 15 to account for clustering at the facility level.

### Qualitative data collection

In December 2017, IDIs and focus groups were conducted with consenting women who had recently delivered in a health facility in Kaltungo and Kwami LGAs. Similar to rest of Gombe State, Kaltungo and Kwami LGAs are multi-ethnic and mostly rural. Public health services account for almost all institutional deliveries in these LGAs, provided through 38 public and six private health facilities in Kaltungo LGA, and 42 public health facilities in Kwami LGA, where there are no private health facilities. Recruitment was purposeful and involved the lead researcher with the assistance of health facility staff identifying women who had delivered in a health facility, from the records of two health facilities in Kaltungo LGA and two health facilities in Kwami LGA, and whose infants were under 6-months of age. The homes of 64 eligible women were then identified with the assistance of community leaders specifically ward focal persons.

The IDIs were conducted in the two most commonly spoken local languages in Gombe, Hausa and Fulfulde, and in English, using a pretested semi-structured interview guide. Out of the 64 women identified and recruited, 31 women – 15 in Kaltungo and 16 in Kwami – were interviewed in-depth before sufficient saturation level was achieved, determined in reflective meetings in-between interviews [39]. One of the women recruited for the IDIs in Kaltungo could not participate because she travelled out of town before the interview. The interviews were conducted in the women’s homes or in a private place of their choosing, by a trained female interviewer. Interview sessions lasted between 60 and 90 min, and were audio recorded, the research lead ensured the quality of the data collected from the IDIs through reflective meetings with the interviewer between the interviews. The interviews focused on problems faced by pregnant women, their motivations for giving birth in a health facility and for selecting a particular health facility, their attitudes towards health facility deliveries, their perceptions of quality of care and experience of mistreatment during institutional delivery.

The IDIs were followed by focus group discussions (FGDs) to gain further insights into the women’s shared understandings of respectful care during institutional delivery and to gain group consensus around themes identified in the IDIs as reasons for giving birth in a health facility and perceptions of quality of care, including respectful care received. Four FGDs were conducted, two in Kwami LGA and two in Kaltungo LGA, with the remaining 32 women from the 64 originally identified. Eight women participated in each FGD with no stratification. For optimal results, 6–12 participants per focus group were recommended [40, 41]. The FGDs were also conducted by a trained female interviewer, assisted by the research lead. To encourage participants to speak freely the FGDs were conducted in neutral settings: empty primary school classrooms and community centre conference rooms. The FGD sessions lasted between 90 and 120 min [42]. The same trained female interviewer carried out both the IDIs and FGDs. IDI and FGD guides were used to ensure that all relevant issues were covered. Data collection took the form of field notes, supported by recordings. At the end of each data collection session, the sound recordings, field notes and consent forms, were stored securely.

### Qualitative data analysis

The recorded qualitative interviews were transcribed verbatim and translated from Hausa or Fulfulde into English. To ensure that the original meanings conveyed by the participants were fully captured the data collectors also carried out the translations. A thematic content analysis a form of qualitative analysis that allows the use of quantitative results as the basis for a priori themes was used to analyse the data, with a manifest approach [40], in which the data analysis focused on what women said of their experience during labour and delivery. The data analysis was carried out in three stages. First, familiarisation involving reading and re-reading the transcripts to aid understanding of the data. Second, organising and coding the data. The coding was determined a priori to align the qualitative findings to the quantitative results, to aid understanding how the quantitative findings were manifest. These were physical abuse, verbal abuse, sexual abuse, stigma and discrimination, failure to meet professional standards of care, poor rapport between women and providers and health system conditions and constraints. The coding was done using NVivo software version 12. Third, data from each code point were reviewed and summarised to reduce the number of words without losing the content or context of the text and to ensure themes were internally consistent. The a priori themes helped in identifying broad initial themes, further themes that emerged from the analysis were considered sub-divisions of those broad initial themes, but may also be standalone. The qualitative study findings were drawn from individual themes and sub-themes, and from exploring the relationship between themes. Some representative anonymised quotes of women’s own words were used to describe the manifestations of their experiences. The credibility of the data was determined by triangulating data between data collection methods.

## Results

### Characteristics of women

The majority of exit interview participants were multigravidae between the ages of 20 and 29 years, from the Fulani or Hausa ethnic groups, nearly all were Muslims, all were married, and half had received no formal education. All women had been attended by female health staff, just 20% of whom were doctors, nurses or midwives. Around 60% of births occurred during the daytime and on a weekday (Table 1).

**Table 1:** Socio-demographic characteristics of exit interview participants and delivery context

	*N* = 342
Characteristics & delivery context	
Age	
< 20 years	17%
20–29 years	57%
30–39 years	23%
40–49 years	3%
Ethnicity	
Fulani	60%
Hausa	20%
Kanuri	6%
Others	14%
Religion	
Christian	2%
Muslim	98%
Marital status	
Married	100%
Single/Widowed	0%
Education level	
None	49%
Primary	20%
Secondary & Post-secondary	31%
Parity	
Primigravida	2%
Multigravida	98%
Supply side characteristics during delivery	
Sex of birth attendants	
Female	100%
Male	0%
Category of birth attendant	
Skilled attendants (doctors, nurses, midwives) formally employed	20%
Non-skilled attendants	80%
Period of birth	
Day time (7.00 am to 7.59 pm)	60%
Night time (8.00 pm to 6.59 am)	40%
Day of birth	
Weekdays	69%
Weekend	31%

Thirty-one women participated in the IDIs; mostly multigravidae between the ages of 20 and 29 years, mostly Muslims, mostly married and nearly half of whom had received no formal education. A total of 32 women participated in FGDs; again the majority being multigravidae and aged 20–29 years, from the Kanuri and Hausa ethnic groups, the majority were Muslims and married, and more than half had received no formal education (Table 2). All had recently delivered in a health facility, the median age of their infants being 6-months at the time of interview.

**Table 2:** Socio-demographic characteristics of the in-depth interviews and focus groups discussion participants

Characteristics	In-depth Interviews	Focus Groups	All Participants
	*N* = 31	*N* = 32	*N* = 63
Age			
< 20 years	29%	9%	19%
20–29 years	48%	50%	49%
30–39 years	19%	41%	30%
40–49 years	3%	0%	2%
Ethnicity			
Fulani	19%	9%	14%
Hausa	13%	19%	16%
Kanuri	10%	13%	11%
Others	58%	59%	59%
Religion			
Christian	35%	28%	32%
Muslim	65%	72%	68%
Marital status			
Married	84%	94%	89%
Single/Widowed	16%	6%	11%
Education level			
None	45%	63%	54%
Primary	10%	3%	6%
Secondary & Post-secondary	45%	34%	40%
Parity			
Primigravida	32%	16%	24%
Multigravida	68%	84%	76%

### Reported prevalence and manifestation of respectful maternity care practices during institutional birth

Quantitative data on prevalence of respectful maternity care practices are presented below according to the following dimensions: (a) health system conditions and constraints, (b) poor rapport between women and providers, (c) failure of professional standards of care, (d) physical abuse, (e) verbal abuse, (f) sexual abuse and (g) stigma and discrimination. Qualitative data are presented alongside these dimensions to highlights the manifestation of respectful maternity care practices during institutional delivery. No clear pattern emerged to indicate differences in the manifestations of mistreatment between Kwami LGA and Kaltungo LGA.

#### Health system conditions and constraints

During exit interviews, cases of mistreatment relating to health system conditions and constraints were reported in up to half of all deliveries 50% (95% CI 31–70). Feeling unclear about the fee structure and health workers making unreasonable requests were reported in 19% (95% CI 7–44) and 19% (95% CI 7–44) of deliveries respectively. In as many as one in four deliveries, respondents reported staffing shortages 24% (95% CI 11–44) and the poor physical condition of health facilities 27% (95% CI 12–49).

The qualitative data supported the high prevalence of mistreatment related to health system conditions and constraints reported in exit interviews. Participants in in-depth-interviews highlighted the different manifestations of health system constraints, which were further confirmed in the FGDs, including women going to the health facility and not getting care because the staff were not available, or being asked to leave the facility to buy delivery materials (e.g. hand gloves, injections, soap or blade) before they were attended to, or being denied attention because they did not have the money to pay, for example, being denied an intravenous infusion when needed, for lack of money. Women expressed discontent at being asked to fetch water or to clean the delivery room themselves before leaving, being asked to deliver on an uncleaned bed with a clear indication of someone else’s blood, delivering on the floor due to lack of beds, feeling uncomfortable due to lack of a fan. Women were unhappy with the unclear fee structure at the health facility, a prevalent feeling among them was that charges for delivery were inconsistent and unjustified. Similarly, women felt the number of items demanded by birth attendants was unnecessary (e.g. two bars of soap, kerosene, or bleach). The presence of too many mosquitos in the delivery room, a dirty environment, or being left exposed with no privacy during birth were also mentioned. Less common manifestations in this dimension included experience of extortion relating to not being attended to without paying what the health workers asked. Consensus among FGD participants was that these constraints inclined some women to opt for home delivery or go to a private clinic if they had the money.

*“The doctor prescribed some medicine for me, he calculated the money more than five times, he will calculate and calculate again with his calculator from 700 to 1500 and another 250 Naira, from there I know there was a problem...” (IDI participant, 32* years, #201*)*

*“We were a lot that day, and this one will deliver that one will deliver, there was no privacy, we were looking at each other just like how baby worms are delivered.” (IDI participant, 30* years, #205*)*

*“…after you have delivered they should cover you, but … I swear that is how they leave you naked …when a woman delivers she needs some privacy, being naked is not good, the angels of mercy will not come over you when you are naked. It is true.” (IDI participant, 32* years, #107*)*

#### Poor rapport between women and providers

From the quantitative data, instances of mistreatment relating to the poor rapport between women and health workers were the second most prevalent dimension with 46% of women reporting such experiences (95% CI 24.4–68.6). Denial or lack of a birth companion during labour and delivery was the most prevalent example of poor rapport between women and providers 28% (95% CI 14–49), followed by lack of supportive care from health workers 18% (95% CI 6–43). Poor communication with the birth attendant during labour and delivery was reported by 15% (95% CI 5–37) of the women.

Women in the IDIs, corroborated those in FGDs, who said they had experienced a poor rapport with a birth attendant and described ineffective communication, such as questions or concerns being ignored, not being informed of what was going to happen to them or their babies, not being informed of test results, health workers discussing the condition of a woman in labour in English knowing she was concerned but could not understand what was being said, or not being received with open arms on arrival. Women further described lack of supportive care as including birth attendants not helping the delivery process, not being sympathetic, caring or kind, being unfriendly for no reason, or not being supportive, for example chasing women away from the facility who did not have delivery items such as gloves. Women lamented their loss of autonomy during labour and delivery, including not being allowed to eat, drink or scream, not being allowed to go to the toilet, asked to urinate or defecate on the delivery bed with no explanation; forced to deliver in a position they were not used to, i.e. lying down instead of squatting. They described feelings of constraint or loneliness because they were not allowed to move around during labour, or their birth companion was not allowed to stay with them – even though the health workers stayed outside and there was no one else in the delivery room. Yet having language and interpretation issues or being denied food, fluids or mobility during labour and delivery were not as frequently reported in exit interviews, at 3% (95& CI 1–5) and 4% (95% CI 1–18) respectively.

Women questioned the value of institutional delivery in the face of such treatment, and in anticipation of such things happening to them again. Mechanisms to minimise their discomfort included having a strong preference for a short stay in a health facility – from labour to delivery to discharge – or praying that a particular staff member would not to be on shift.

*“I will get up and squat, and they will say no, I should lie down, I will get up again and sit down and she [birth attendant] will say no, lie down. I did not know how they give birth in a facility [my first time] …I have never heard of it, I swear I have never heard of giving birth lying down.” (IDI participant, 30* years, #206*)*

*“…exactly, you can hear women saying I pray I don’t meet madam so and so today in the facility because of her attitude.” (FGD participant, 35* years, #102_4*)*

*“…they [health workers] asked, was it a delivery and I said yes, she [health worker] said was it my first and I said yes again, she took me in, checked and said the baby was not due yet, she was rude, …and went out to continue talking with her friends. Whenever I tell her I need something like going to pee she wouldn’t even talk to me. But the other nurse that came later sat with me and was talking to me about what to do. I felt like I should have delivered at home. I even told my mother to move me [to a different hospital] before the other nurse came in.” (IDI participant, 17* years, #106*)*

The women hoped for birth attendants to receive them with open arms when they reached the health facility, for birth attendants to be supportive during labour by being helpful, kind and encouraging to women when in labour pains. They hoped for effective communication and some autonomy. Women that experienced such a positive rapport and interaction with health workers expressed happiness with their facility birth experience. Some women even suggested not minding being denied a birth companion or preferred birth position, as long as the birth attendant was supportive through the delivery.

*“She [birth attendant] said I should lay down and she sat beside me, later on when the labour pain started again, she came closer to me and asked me to breathe while she was bringing out her hand gloves and all other things she will need for the delivery. After a while, the baby’s head came out, and she helped me to bring him out. She was kind to me because when I was crying, she told me not to cry it will soon be over and I will rest.” (FGD participant, 29* years, #102_5*)*

#### Failure to meet professional standards of care

Forty-four percent (95% CI 24–66) of the women reported experiencing incidences of mistreatment relating to failure to meet professional standards of care. The most commonly reported being a lack of informed consent processes, 25% of women (95% CI 11–47). Absence of skilled attendant at the time of delivery, 18% (95% CI 6–32), and painful vaginal examination, 18% (95% CI 7–41), were also commonly reported.

While only 8% (95% CI 5–13) of women reported neglect, abandonment or long delays in the exit interviews, in IDIs and FGDs women described profound displeasure at being neglected even though they were in a health facility, being ignored while they needed help, being abandoned to deliver alone while in a health facility and experiencing long delays before receiving attention. Women highlighted experiencing episiotomy without pain relief – even when they expressed pain, or not being asked for consent before procedures. Other important manifestation was the absence of skilled staff, with cleaners and non-clinical staff conducting deliveries. Women freely described their displeasure in relation to this dimension, they did not offer any defence of health worker behaviour or the health system, and did not blame themselves for receiving such mistreatment and saw these as strong reasons for causing reluctance to deliver in a health facility again.

*“It had happened to me during my first delivery before the nurse came, the cleaner did everything to me [conducted the delivery].” (FGD participant, 24* years, #101_8*)*

*“The baby came out with the cord around her neck, so I struggled to remove it off her [on my own]. Before she [health worker] could come to me, I had already removed the cord that was around the baby’s neck.” (FGD participant, 28* years, #201_2*)*

*“I had a tear [during delivery], they [health workers] just started the episiotomy without any injection [for pain relief] while they knew that it was a painful procedure, I was not happy at all. They should inform me what they were going to do to have my consent, I think that would have been the right thing to do.” (IDI participant, 24* years, #102*)*

#### Physical abuse

From the exit survey, just 3% of women (95% CI 2–4) reported being beaten, pushed, slapped or poked during delivery and only 1% (95% CI 0–3) reported experiencing any form of physical restraint (Table 3).

**Table 3:** Self-reported experience of respectful care during institutional childbirth

Main	Manifestations/indicators	*N* = 342
Dimensions		%(95%CI)
Health system conditions and constraints	Poor physical condition of facilities (dirty delivery room)	27 (12–49)
	Staffing shortages (e.g. not enough staff) during labour and delivery	24 (11–44)
	Supply constraints (e.g. essential medicines and supply not available)	13 (7–21)
	Lack of privacy (e.g. feeling of being exposed during delivery)	15 (4–42)
	Lack of redress	5 (2–11)
	Bribery and extortion during facility birth	1 (0–3)
	Feeling of unclear fee structures (e.g. lack of visible price list)	19 (7–44)
	Feeling of unreasonable requests by health workers	19 (7–44)
	Experience of any abuse related to health system conditions and constraints	50 (31–70)
Poor rapport between women and providers	Poor communication with the BA during labour and delivery	15 (5–37)
	Dismissal of woman (companion) concerns during labour and delivery	7 (3–14)
	Had language and interpretation issues during labour and delivery	3 (1–5)
	Poor staff attitudes during labour and delivery	9 (5–16)
	Lack of supportive care from health workers	18 (6–43)
	Denial or lack of birth companions during labour and delivery	28 (14–49)
	Being treated as passive participants during childbirth	7 (2–27)
	Denial of food, fluids, or mobility during labour and delivery	4 (1–18)
	Lack of respect for preferred birth positions	13 (8–22)
	Denial of safe traditional practices	5 (1–15)
	Detained in a facility for failure to pay for services	6 (1–21)
	Experience of any abuse related to poor rapport between women and providers	46 (24–69)
Failure to meet professional standards of care	Lack of informed consent process (e.g. examine without permission)	25 (11–47)
	Breach of confidentiality (women private information shared)	1 (1–2)
	Painful vaginal exams (refusal to provide pain relief)	18 (7–41)
	Neglect, abandonment, or long delays (Ignored when help is needed)	8 (5–13)
	Skilled attendant absent at time of delivery	18 (6–32)
	Performance of unconsented surgical operations	2 (1–7)
	Experience of any abuse related to failure to meet professional standards of care	44 (24–66)
Physical abuse	Being beaten, pushed, pinched, slapped or poked	3 (2–4)
	Physically restrained, tied or gagged during labour and delivery	1 (0–3)
	Experience of any physical abuse	3 (2–5)
Verbal abuse	Harsh or rude language, judgmental or accusatory comments	10 (6–18)
	Threats of withholding treatment or blamed for poor birth outcomes	6 (3–12)
	Experience of any verbal abuse	11 (6–20)
Sexual abuse	Sexual abuse touched inapproachably or raped	0
	Any sexual abuse	0
Stigma & discrimination	Discriminated against based on ethnicity, religion, income, disease	0
	Experience of any abuse related to discrimination	0
Experience of any abuse		66 (45–82)

According to women in the IDIs and FGDs, use of force was primarily manifested as women being slapped or hit during labour and delivery, with tying of legs (apart) as the main form of restraint, although respondents were of the view that this rarely occurs in their setting. Rather, they felt that physical abuse of this kind was limited to a few specific health workers and done to exert compliance or obedience. Where it had been experienced, women reported dealing with physical abuse in different ways, for example, by mostly ignoring the mistreatment, focusing on their goal to deliver safely and receiving the help that took them to the hospital in the first place. One woman reported fighting back. A commonly held view among women in IDIs and FGDs was that the blame lay with the women in labour for instigating the slapping, hitting or tying of legs, rather than with the health workers. Physical abuse did not seem to be a deterrent for choosing to have a subsequent birth at a health facility. Women reported being able to identify an abusive health worker from antenatal care visits and subsequently choosing to go to a different facility to avoid her or him.

*“I was not married [young] when I had my first birth, you know there was shyness at that age. I went to deliver with my pants on, the nurse asked me to pull my pants down, but I was reluctant. She [Nurse] insisted resulting with the two of us both pulling at my pant, that was when she slapped me.” (IDI participant, 24* years, #103*)*

*“They usually slap women during delivery because they might ask you to do something if you refused to comply because of the labour pains they will slap you. That is their work, and maybe you didn’t behave well.” (IDI participant, 20* years, #202*)*

*“Though I was not happy, I have no option than to come back [to the health facility]. She [a particular health worker] is always the talk of the village, nobody is happy about her, she is not nice, only she behaves that way [shouting or hitting], but the others [health workers] are good.” (IDI participant, 25* years, #108*)*

#### Verbal abuse

In the exit interviews, 11% (95% CI 6–20) of women reported experiencing forms of verbal abuse. Use of harsh, rude or judgemental language or comments were experienced by 10% (95% CI 6–18) of women, while 6% (95% CI 3–12) reported being threatened and or blamed during labour and delivery.

A common manifestation of verbal abuse recounted by women in IDIs and reinforced in FGDs, included health care workers being unfriendly, shouting or scolding women. Forms of threats and blame manifested as being blamed for poor childbirth outcomes, or being rushed, for instance; asked to hurry and deliver, or being left alone with no attention during labour. In the FGDs women expressed frustration at being verbally assaulted in addition to their labour pains. Some tried to justify frequent verbal assaults by health workers during labour or delivery, attributing the verbal abuse to the wailing and screaming of women during delivery. Despite verbal abuse, nearly all the women in IDIs and FGDs described the medication or drugs, injections and assistance from the health workers as sufficient motivation to deliver in a health facility again, with just a few saying it would be better to deliver at home with dignity. Suggested mechanisms to avoid verbal abuse included obeying all orders by health care workers, going to the health facility prepared with all the required birth items, e.g. gloves, pampers or baby napkins, attending antenatal care and being patient with labour pains, i.e. not crying or shouting.

*“They have never done it to me, but I once escorted a woman to the home. [After a prolonged labour] she gave birth, but the baby has already died in her stomach [still birth]. You know delivering a live baby and the baby that is lifeless is different because a live baby helps you in the process of coming out, unlike the one that is lifeless…they kept telling her…that she was the one that killed her baby…that she is used to doing that every time, that her babies don’t come out alive. Honestly, I was angry with them.” (IDI participant, 32* years, #201*)*

#### Sexual abuse

Questions around experience of sexual abuse were adapted for the Gombe context to include touching the sexual organs of a woman in labour with sexual intent, gestures suggestive of sexual interest, selective attention suggestive of sexual interest, or rape. No women reported experiencing sexual abuse.

Similarly, none of the respondents from the FGDs or IDIs could describe a sexual abuse incident, either based on their own or another’s experience. An FGD participant described her discomfort at being attended by a male birth attendant, which she found unsettling, she described not being able to go back to the same facility for a long time. An opinion shared by many in the group but not all. In general, women were uncomfortable talking about the subject of sexual abuse.

“*You know women don’t want a male health care worker to conduct their delivery. One will prefer a female to do that. If a male health worker is the one that conducted your delivery, you would not feel comfortable anytime you see him. This can make one to either deliver at home or move to another facility…since my delivery, anytime I go to the health facility and see him [the male health worker] I feel uncomfortable.” (FGD participant, 37* years, #201_4*)*

#### Stigma and discrimination

Only one woman among the exit interviewees reported experiencing an act, a gesture or being treated negatively because of her tribe, religion, socioeconomic status, disease condition, or age (0.3%).

Differing opinions were expressed during qualitative interviews (IDIs and FGDs), again with the majority of women talking about stigma and discrimination they had witnessed, rather than what they had experienced themselves. Manifestations highlighted included women from the city being given preferential treatment over women from the village, those considered rich treated better than those considered poor, and people from the same religion or tribe as the health care worker being treated preferentially. Women also described being treated negatively when they went to health care facilities outside their community, catchment areas or LGA.

*“Yes, they discriminate, especially to a village person. I swear, they show discrimination. You cannot say you are from the village without them [health workers] giving you a nasty look, dust and scorn.” (IDI participant, 20* years, #206*)*

*“...it was due to the lack of medical attention here in the health facility serving our community that made her go to Bula hospital [health facility serving a different community]. The health workers there did not receive us well, and they said why don’t we go to the facility in our community for treatment, why disturb them here?” (FGD participant, 28* years, #201_8*)*

However, some participants insisted that such practices do not occur in their facilities.

*“The hospital where I went there was no discrimination. Even though our tribe and religion were not the same [as the health workers], but honestly, …they even treat us better than the ones [health workers] who are of the same tribe and religion as us. We enjoyed going there, there was no argument.” (IDI participant, 32* years, #207*)*

## Discussion

Mistreatment was reported in 66% of all institutional births, with reported prevalence varying across the dimensions of care. Women’s reported experience of mistreatment predominantly arose because of health system conditions and constraints (50% of all women) and instances of mistreatment related to a poor rapport between women and providers (46% of all women). Mistreatment related to sexual abuse, stigma and discrimination were reported the least. Qualitative findings highlighted different forms of mistreatment that might take place during institutional births and that mistreatment could affect subsequent decisions about where to deliver.

Our findings of frequent mistreatment during institutional delivery in northeast Nigeria are consistent with findings both from other low and middle- income settings [2, 16–18, 20, 21, 23, 38–46] and from other regions in Nigeria [47–51], underscoring the need for country and global advocacy to address women’s experiences of care during institutional delivery [12, 52]. However, the different dimensions of abuse were not consistently reported between settings. In different settings in Nigeria for example, reported prevalence of physical abuse ranged from 2 and 36% (3% in this study), discrimination from 0 and 20% (0% in this study) and neglect and abandonment ranged from 8 and 24% (8% in this study) [24]. This emerging pattern of high frequency of negative experience but heterogeneity in the dimensions, suggests that strategies for improvement need to take account of contextual differences [2]. The qualitative data further emphasised some of these nuances, revealing women value being received with open arms when they first arrive at a health facility and being supported, treated kindly and encouraged through the birthing process. They appreciate when their choices and concerns are considered, and when a birth attendant communicates with them effectively. Moreover, a unique manifestation of mistreatment from the qualitative findings concerned women being discriminated against when they deliver in health facilities outside their catchment area or communities, underlining what matters and what works may differ from one setting to another.

Participants in the qualitative interviews were likely to have attended any of the ten primary health care facilities. They were selected to describe how mistreatment occurs in Gombe State. Although the exit interviewees were more likely to be multigravida, married, Muslims and Fulani’s than the qualitative interviewees, we do not expect this to have changed the conclusions arising from this study, as the results are supposed to be complimentary, not to converge to provide the same conclusions [53]. This study provided complimentary results relating to two different aspects of mistreatment: (1) the frequency with which mistreatment occurs in Gombe state, and (2) the presentation of mistreatment when it occurs [53].

### Implications for quality of care

This study presents the first evidence of mistreatment during institutional delivery in Gombe State. Mistreatment appears to be a deterrent to utilisation of MNH services in Gombe. These findings taken together with findings from other settings in Nigeria may be an indication of why delivery at home continues to be a more attractive option for women in Nigeria. Women are aware of the possibility of going to the facility and not meeting a health worker, and that even if they do, the health worker may not be skilled. Women anticipate that the labour and delivery environment may not be ideal (e.g. no water, not clean, disturbed by mosquitos, no screen to provide privacy). They are aware that in addition to surviving the poor state of the health facilities, they may have to cope with one or more overworked and less motivated health workers with a poor attitude.

To improve the birth environment in health facilities in the country, Nigeria is promoting a task-shifting and sharing policy [54]. The task-shifting policy aims to improve access to skilled birth attendants and to address the health worker shortages especially at PHCs where about 90% of deliveries are conducted by lower cadre health workers (i.e. CHEW, JCHEWS). One specific objective of the task-shifting policy is to train the CHEWs to a level that they can provide routine maternal and newborn care, basic emergency obstetrics and newborn care, and referral for comprehensive emergency obstetric and newborn care when needed [54]. Studies on the experience of care in Nigeria, including findings from this study, suggest the need to emphasise respectful maternity care in the task-shifting policy recommendations and curriculum for frontline health care workers in Gombe State and Nigeria as a whole. So that when CHEWS are the only birth attendant available in a health facility, as was the case in our study sites, they can provide quality care both in terms of content and experience of care. The Community Health Influencers, Promoters, and Services programme (CHIPS), is another initiative of the federal government of Nigeria, designed to link communities with the health system through community outreach [55]. The CHIPS program could be utilised in Gombe and in Nigeria to also educate communities about women’s rights to respectful maternity care.

We found that the incidence of mistreatment due to health system conditions and constraints appeared to be a strong deterrent to the subsequent use of health facilities for delivery. Under-resourced and strained health systems have been associated with increased incidence of disrespect and abuse in other settings [48, 49, 56] and consequently the delayed utilization or non-utilization of institutional delivery services [5, 51, 61, 58]. Accordingly, interventions that seek to address mistreatment in the study setting and other similar settings, should emphasize addressing system-wide constraints [20, 59–61]. For example, when two or three women seek delivery care simultaneously, at a health centre with only two rooms for inpatient and outpatient services and only two health staff on duty, those women are likely to experience forms of mistreatment aligned with health system constraints. In this scenario, women may experience a lack of privacy in the delivery room, or a lack of prompt attention due to shortages of health care workers.

Addressing system-wide constraints is challenging in resource-limited settings such as Gombe, but possible. For example, stakeholders in Gombe including policymakers, health care providers, women and communities, could come together to review these findings, negotiate standards of care for labour and childbirth [62] and agree on contextually appropriate ways to institutionalise the agreed standards in health facilities to improve women’s experience of care. Such an approach has already been successfully tried in two settings of Nigeria [62] and is worth considering in Gombe and other similar settings.

### Strengths and limitations

This study provides a rich description of the frequency of indicators of mistreatment during institutional delivery, and women’s own views about the experience of care. Nonetheless it has limitations. The exit interviews were conducted within health facilities, a possible source of courtesy bias [19]. To minimise this, respondents were interviewed away from the facility staff and assured anonymity and confidentiality [63]. Additionally, we excluded women discharged without a live baby, which had the potential to affect our estimates due to differential risks for reporting mistreatment [19]. The health facilities were not selected to represent the state, limiting generalisability. Our study tool was based on women’s self-report of perceived mistreatment, which may not be an accurate reflection of all mistreatment, and sample size restrictions limited any tests for association. Nonetheless, the study provided evidence of prevalence of mistreatment during institutional delivery in Gombe State that was previously lacking. The exit interviews were conducted within 24 h post-partum and women’s reflections on the birthing experience may be different to women interviewed later after birth [64, 65]. In some studies, exit interviews have been complimented with community interviews with women 4–10 weeks post-delivery to compare what women reported at exit [64, 66]. Our qualitative sample comprised of women whose infants were about 6-months old, meaning that they had more time to reflect on their facility birth experience. However, the two data sources were designed to be complementary, with the qualitative data further elaborating and illustrating the manifestations of mistreatment.

## Conclusions

Our study showed that mistreatment during institutional delivery is frequent in northeast Nigeria and risks jeopardising efforts to increase the coverage of institutional deliveries. To address this problem, immediate and sustained attention to the quality of care as it pertains to the experience of care users is needed.

## Supplementary information

**Additional file 1: Table S1.** Mistreatment questions.

## Data Availability

The datasets used and/or analysed during the current study are available from the corresponding author on reasonable request.

## Abbreviations

CI: Confidence Interval
COREQ: Consolidated Criteria for Reporting Qualitative Research
CSPro: Census and Survey Processing System
FGDs: Focus Group Discussions
IDIs: In-depth interviews

## References

[1] Tunçalp Ӧ, Were W, MacLennan C, Oladapo O, Gülmezoglu A, Bahl R (2015). Quality of care for pregnant women and newborns-the WHO vision. BJOG An Int J Obstet Gynaecol.

[2] Banks KP, Karim AM, Ratcliffe HL, Betemariam W, Langer A (2018). Jeopardizing quality at the frontline of healthcare: prevalence and risk factors for disrespect and abuse during facility-based childbirth in Ethiopia. Health Policy Plan.

[3] Montagu D, Sudhinaraset M, Diamond-Smith N, Campbell O, Gabrysch S, Freedman L (2017). Where women go to deliver: understanding the changing landscape of childbirth in Africa and Asia. Health Policy Plan.

[4] National Population Commission (NPC) [Nigeria] and ICF (2019). Nigeria Demographic and Health Survey 2018 Key Indicators Report.

[5] Ashimi AO, Amole TG (2015). Prevalence, reasons and predictors for home births among pregnant women attending antenatal care in Birnin kudu, North-west Nigeria. Sex Reprod Healthc.

[6] Okonofua F, Ntoimo L, Ogungbangbe J, Anjorin S, Imongan W, Yaya S (2018). Predictors of women’s utilization of primary health care for skilled pregnancy care in rural Nigeria. BMC pregnancy childbirth. BMC Pregnancy and Childbirth.

[7] Bohren MA, Hunter EC, Munthe-Kaas HM, Souza JP, Vogel JP, Gülmezoglu AM. Facilitators and barriers to facility-based delivery in low- and middle-income countries: a qualitative evidence synthesis. Reprod Health. 2014. 10.1186/1742-4755-11-71. 10.1186/1742-4755-11-71 PMC4247708 25238684

[8] Kassebaum Nicholas J, Bertozzi-Villa Amelia, Coggeshall Megan S, Shackelford Katya A, Steiner Caitlyn, Heuton Kyle R, Gonzalez-Medina Diego, Barber Ryan, Huynh Chantal, Dicker Daniel, Templin Tara, Wolock Timothy M, Ozgoren Ayse Abbasoglu, Abd-Allah Foad, Abera Semaw Ferede, Abubakar Ibrahim, Achoki Tom, Adelekan Ademola, Ademi Zanfina, Adou Arsène Kouablan, Adsuar José C, Agardh Emilie E, Akena Dickens, Alasfoor Deena, Alemu Zewdie Aderaw, Alfonso-Cristancho Rafael, Alhabib Samia, Ali Raghib, Al Kahbouri Mazin J, Alla François, Allen Peter J, AlMazroa Mohammad A, Alsharif Ubai, Alvarez Elena, Alvis-Guzmán Nelson, Amankwaa Adansi A, Amare Azmeraw T, Amini Hassan, Ammar Walid, Antonio Carl A T, Anwari Palwasha, Ärnlöv Johan, Arsenijevic Valentina S Arsic, Artaman Ali, Asad Majed Masoud, Asghar Rana J, Assadi Reza, Atkins Lydia S, Badawi Alaa, Balakrishnan Kalpana, Basu Arindam, Basu Sanjay, Beardsley Justin, Bedi Neeraj, Bekele Tolesa, Bell Michelle L, Bernabe Eduardo, Beyene Tariku J, Bhutta Zulfiqar, Bin Abdulhak Aref, Blore Jed D, Basara Berrak Bora, Bose Dipan, Breitborde Nicholas, Cárdenas Rosario, Castañeda-Orjuela Carlos A, Castro Ruben Estanislao, Catalá-López Ferrán, Cavlin Alanur, Chang Jung-Chen, Che Xuan, Christophi Costas A, Chugh Sumeet S, Cirillo Massimo, Colquhoun Samantha M, Cooper Leslie Trumbull, Cooper Cyrus, da Costa Leite Iuri, Dandona Lalit, Dandona Rakhi, Davis Adrian, Dayama Anand, Degenhardt Louisa, De Leo Diego, del Pozo-Cruz Borja, Deribe Kebede, Dessalegn Muluken, deVeber Gabrielle A, Dharmaratne Samath D, Dilmen Uğur, Ding Eric L, Dorrington Rob E, Driscoll Tim R, Ermakov Sergei Petrovich, Esteghamati Alireza, Faraon Emerito Jose A, Farzadfar Farshad, Felicio Manuela Mendonca, Fereshtehnejad Seyed-Mohammad, de Lima Graça Maria Ferreira, Forouzanfar Mohammad H, França Elisabeth B, Gaffikin Lynne, Gambashidze Ketevan, Gankpé Fortuné Gbètoho, Garcia Ana C, Geleijnse Johanna M, Gibney Katherine B, Giroud Maurice, Glaser Elizabeth L, Goginashvili Ketevan, Gona Philimon, González-Castell Dinorah, Goto Atsushi, Gouda Hebe N, Gugnani Harish Chander, Gupta Rahul, Gupta Rajeev, Hafezi-Nejad Nima, Hamadeh Randah Ribhi, Hammami Mouhanad, Hankey Graeme J, Harb Hilda L, Havmoeller Rasmus, Hay Simon I, Pi Ileana B Heredia, Hoek Hans W, Hosgood H Dean, Hoy Damian G, Husseini Abdullatif, Idrisov Bulat T, Innos Kaire, Inoue Manami, Jacobsen Kathryn H, Jahangir Eiman, Jee Sun Ha, Jensen Paul N, Jha Vivekanand, Jiang Guohong, Jonas Jost B, Juel Knud, Kabagambe Edmond Kato, Kan Haidong, Karam Nadim E, Karch André, Karema Corine Kakizi, Kaul Anil, Kawakami Norito, Kazanjan Konstantin, Kazi Dhruv S, Kemp Andrew H, Kengne Andre Pascal, Kereselidze Maia, Khader Yousef Saleh, Khalifa Shams Eldin Ali Hassan, Khan Ejaz Ahmed, Khang Young-Ho, Knibbs Luke, Kokubo Yoshihiro, Kosen Soewarta, Defo Barthelemy Kuate, Kulkarni Chanda, Kulkarni Veena S, Kumar G Anil, Kumar Kaushalendra, Kumar Ravi B, Kwan Gene, Lai Taavi, Lalloo Ratilal, Lam Hilton, Lansingh Van C, Larsson Anders, Lee Jong-Tae, Leigh James, Leinsalu Mall, Leung Ricky, Li Xiaohong, Li Yichong, Li Yongmei, Liang Juan, Liang Xiaofeng, Lim Stephen S, Lin Hsien-Ho, Lipshultz Steven E, Liu Shiwei, Liu Yang, Lloyd Belinda K, London Stephanie J, Lotufo Paulo A, Ma Jixiang, Ma Stefan, Machado Vasco Manuel Pedro, Mainoo Nana Kwaku, Majdan Marek, Mapoma Christopher Chabila, Marcenes Wagner, Marzan Melvin Barrientos, Mason-Jones Amanda J, Mehndiratta Man Mohan, Mejia-Rodriguez Fabiola, Memish Ziad A, Mendoza Walter, Miller Ted R, Mills Edward J, Mokdad Ali H, Mola Glen Liddell, Monasta Lorenzo, de la Cruz Monis Jonathan, Hernandez Julio Cesar Montañez, Moore Ami R, Moradi-Lakeh Maziar, Mori Rintaro, Mueller Ulrich O, Mukaigawara Mitsuru, Naheed Aliya, Naidoo Kovin S, Nand Devina, Nangia Vinay, Nash Denis, Nejjari Chakib, Nelson Robert G, Neupane Sudan Prasad, Newton Charles R, Ng Marie, Nieuwenhuijsen Mark J, Nisar Muhammad Imran, Nolte Sandra, Norheim Ole F, Nyakarahuka Luke, Oh In-Hwan, Ohkubo Takayoshi, Olusanya Bolajoko O, Omer Saad B, Opio John Nelson, Orisakwe Orish Ebere, Pandian Jeyaraj D, Papachristou Christina, Park Jae-Hyun, Caicedo Angel J Paternina, Patten Scott B, Paul Vinod K, Pavlin Boris Igor, Pearce Neil, Pereira David M, Pesudovs Konrad, Petzold Max, Poenaru Dan, Polanczyk Guilherme V, Polinder Suzanne, Pope Dan, Pourmalek Farshad, Qato Dima, Quistberg D Alex, Rafay Anwar, Rahimi Kazem, Rahimi-Movaghar Vafa, ur Rahman Sajjad, Raju Murugesan, Rana Saleem M, Refaat Amany, Ronfani Luca, Roy Nobhojit, Pimienta Tania Georgina Sánchez, Sahraian Mohammad Ali, Salomon Joshua A, Sampson Uchechukwu, Santos Itamar S, Sawhney Monika, Sayinzoga Felix, Schneider Ione J C, Schumacher Austin, Schwebel David C, Seedat Soraya, Sepanlou Sadaf G, Servan-Mori Edson E, Shakh-Nazarova Marina, Sheikhbahaei Sara, Shibuya Kenji, Shin Hwashin Hyun, Shiue Ivy, Sigfusdottir Inga Dora, Silberberg Donald H, Silva Andrea P, Singh Jasvinder A, Skirbekk Vegard, Sliwa Karen, Soshnikov Sergey S, Sposato Luciano A, Sreeramareddy Chandrashekhar T, Stroumpoulis Konstantinos, Sturua Lela, Sykes Bryan L, Tabb Karen M, Talongwa Roberto Tchio, Tan Feng, Teixeira Carolina Maria, Tenkorang Eric Yeboah, Terkawi Abdullah Sulieman, Thorne-Lyman Andrew L, Tirschwell David L, Towbin Jeffrey A, Tran Bach X, Tsilimbaris Miltiadis, Uchendu Uche S, Ukwaja Kingsley N, Undurraga Eduardo A, Uzun Selen Begüm, Vallely Andrew J, van Gool Coen H, Vasankari Tommi J, Vavilala Monica S, Venketasubramanian N, Villalpando Salvador, Violante Francesco S, Vlassov Vasiliy Victorovich, Vos Theo, Waller Stephen, Wang Haidong, Wang Linhong, Wang XiaoRong, Wang Yanping, Weichenthal Scott, Weiderpass Elisabete, Weintraub Robert G, Westerman Ronny, Wilkinson James D, Woldeyohannes Solomon Meseret, Wong John Q, Wordofa Muluemebet Abera, Xu Gelin, Yang Yang C, Yano Yuichiro, Yentur Gokalp Kadri, Yip Paul, Yonemoto Naohiro, Yoon Seok-Jun, Younis Mustafa Z, Yu Chuanhua, Jin Kim Yun, El Sayed Zaki Maysaa, Zhao Yong, Zheng Yingfeng, Zhou Maigeng, Zhu Jun, Zou Xiao Nong, Lopez Alan D, Naghavi Mohsen, Murray Christopher J L, Lozano Rafael (2014). Global, regional, and national levels and causes of maternal mortality during 1990–2013: a systematic analysis for the Global Burden of Disease Study 2013. The Lancet.

[9] Say Lale, Chou Doris, Gemmill Alison, Tunçalp Özge, Moller Ann-Beth, Daniels Jane, Gülmezoglu A Metin, Temmerman Marleen, Alkema Leontine (2014). Global causes of maternal death: a WHO systematic analysis. The Lancet Global Health.

[10] Campbell Oona M R, Calvert Clara, Testa Adrienne, Strehlow Matthew, Benova Lenka, Keyes Emily, Donnay France, Macleod David, Gabrysch Sabine, Rong Luo, Ronsmans Carine, Sadruddin Salim, Koblinsky Marge, Bailey Patricia (2016). The scale, scope, coverage, and capability of childbirth care. The Lancet.

[11] Souza João Paulo, Gülmezoglu Ahmet Metin, Vogel Joshua, Carroli Guillermo, Lumbiganon Pisake, Qureshi Zahida, Costa Maria José, Fawole Bukola, Mugerwa Yvonne, Nafiou Idi, Neves Isilda, Wolomby-Molondo Jean-José, Bang Hoang Thi, Cheang Kannitha, Chuyun Kang, Jayaratne Kapila, Jayathilaka Chandani Anoma, Mazhar Syeda Batool, Mori Rintaro, Mustafa Mir Lais, Pathak Laxmi Raj, Perera Deepthi, Rathavy Tung, Recidoro Zenaida, Roy Malabika, Ruyan Pang, Shrestha Naveen, Taneepanichsku Surasak, Tien Nguyen Viet, Ganchimeg Togoobaatar, Wehbe Mira, Yadamsuren Buyanjargal, Yan Wang, Yunis Khalid, Bataglia Vicente, Cecatti José Guilherme, Hernandez-Prado Bernardo, Nardin Juan Manuel, Narváez Alberto, Ortiz-Panozo Eduardo, Pérez-Cuevas Ricardo, Valladares Eliette, Zavaleta Nelly, Armson Anthony, Crowther Caroline, Hogue Carol, Lindmark Gunilla, Mittal Suneeta, Pattinson Robert, Stanton Mary Ellen, Campodonico Liana, Cuesta Cristina, Giordano Daniel, Intarut Nirun, Laopaiboon Malinee, Bahl Rajiv, Martines Jose, Mathai Matthews, Merialdi Mario, Say Lale (2013). Moving beyond essential interventions for reduction of maternal mortality (the WHO Multicountry Survey on Maternal and Newborn Health): a cross-sectional study. The Lancet.

[12] Kruk Margaret E, Pate Muhammad, Mullan Zoë (2017). Introducing The Lancet Global Health Commission on High-Quality Health Systems in the SDG Era. The Lancet Global Health.

[13] Kruk Margaret E, Gage Anna D, Joseph Naima T, Danaei Goodarz, García-Saisó Sebastián, Salomon Joshua A (2018). Mortality due to low-quality health systems in the universal health coverage era: a systematic analysis of amenable deaths in 137 countries. The Lancet.

[14] Shakibazadeh E, Namadian M, Bohren MA, Vogel JP, Rashidian A, Nogueira Pileggi V, Madeira S, Leathersich S, Tunçalp Ӧ, Oladapo OT, Souza JP, Gülmezoglu AM (2017). Respectful care during childbirth in health facilities globally: a qualitative evidence synthesis. BJOG: An International Journal of Obstetrics & Gynaecology.

[15] Jewkes Rachel, Penn-Kekana Loveday (2015). Mistreatment of Women in Childbirth: Time for Action on This Important Dimension of Violence against Women. PLOS Medicine.

[16] Savage V, Castro A. Measuring mistreatment of women during childbirth: a review of terminology and methodological approaches prof. Suellen Miller. Reprod Health. 2017. 10.1186/s12978-017-0403-5. 10.1186/s12978-017-0403-5 PMC5658997 29073914

[17] Bowser D, Hill K. Exploring evidence for disrespect and childbirth, in facility-based analysis, report of a landscape [Internet]: USAID Traction Project; 2010. Available: https://www.ghdonline.org/uploads/Respectful_Care_at_Birth_9-20-101_Final1.pdf. Accessed 20 Jan 2019

[18] Bohren Meghan A., Vogel Joshua P., Hunter Erin C., Lutsiv Olha, Makh Suprita K., Souza João Paulo, Aguiar Carolina, Saraiva Coneglian Fernando, Diniz Alex Luíz Araújo, Tunçalp Özge, Javadi Dena, Oladapo Olufemi T., Khosla Rajat, Hindin Michelle J., Gülmezoglu A. Metin (2015). The Mistreatment of Women during Childbirth in Health Facilities Globally: A Mixed-Methods Systematic Review. PLOS Medicine.

[19] Sando D, Abuya T, Asefa A, Banks KP, Freedman LP, Kujawski S (2017). Methods used in prevalence studies of disrespect and abuse during facility based childbirth: lessons learned prof. Suellen Miller. Reprod Health.

[20] Ratcliffe HL, Sando D, Lyatuu GW, Emil F, Mwanyika-Sando M, Chalamilla G, et al. Mitigating disrespect and abuse during childbirth in Tanzania: an exploratory study of the effects of two facility-based interventions in a large public hospital. Reprod Health. 2016;13. 10.1186/s12978-016-0187-z. 10.1186/s12978-016-0187-z PMC4948096 27424608

[21] Rosen HE, Lynam PF, Carr C, Reis V, Ricca J, Bazant ES, et al. Direct observation of respectful maternity care in five countries: a cross-sectional study of health facilities in east and southern Africa. BMC Pregnancy Childbirth. 2015;15. 10.1186/s12884-015-0728-4. 10.1186/s12884-015-0728-4 PMC4657214 26596353

[22] Warren C, Njuki R, Abuya T, Ndwiga C, Maingi G, Serwanga J (2013). Study protocol for promoting respectful maternity care initiative to assess, measure and design interventions to reduce disrespect and abuse during childbirth in Kenya. BMC Pregnancy Childbirth..

[23] Abuya T, Ndwiga C, Ritter J, Kanya L, Bellows B, Binkin N, et al. The effect of a multi-component intervention on disrespect and abuse during childbirth in Kenya. BMC Pregnancy Childbirth. 2015. 10.1186/s12884-015-0645-6. 10.1186/s12884-015-0645-6 PMC4580125 26394616

[24] Ishola F, Owolabi O, Filippi V (2017). Disrespect and abuse of women during childbirth in Nigeria: a systematic review. PLoS One.

[25] Federal Ministry of Health (FMoH) (2017). National Healtth Facility Survey [Internet].

[26] National Bureau of Statistics (NBS) and United Nations Children’s Fund (UNICEF) (2018). Multiple Indicator Cluster Survey 2016–17, Survey Findings Report.

[27] National Population Commission (NPC) [Nigeria] and ICF International. Nigeria Demographic and Health Survey 2013. Abuja and Rockville: NPC and ICF International; 2014.

[28] Alkali Y, Jalo I, El–Nafaty A, Bode-Thomas F (2014). Causes of stillbirth in a community survey in Gombe State. Niger J Paediatr.

[29] Basheer Yahya M, Pumpaibool T. Factors affecting women-willingness to pay for maternal, neonatal andChild health services (MNCH) in Gombe state, Nigeria. J Womens Heal Care. 2017;06. 10.4172/2167-0420.1000404.

[30] Yahya MB, Pumpaibool T (2019). Factors influencing the decision to choose a birth center by pregnant women in Gombe state Nigeria: baseline survey. J Heal Res.

[31] National Population Commission of Nigeria. Nigeria Demographic and Health Survey 2013. National Population Commission. 2014. 10.1111/j.1728-4465.2008.00154.x.

[32] Gombe State Government. History of Gombe State, Nigeria 2018 [Internet]. 2018. Available: http://gombestate.gov.ng/history-2. Accessed 20 Jan 2019.

[33] Oruonye ED, Abubakar H, Ahmed MY, Dan Y (2017). HIV/AIDS Interventions in Gombe State Nigeria; Challenges of Sustaining the Gains. Int J Asian Soc Sci.

[34] National Population Commission (NPC) [Nigeria] and ICF. Nigeria Demographic and Health Survey Key Indicators Report. Abuja and Rockville: National Population Commission (NPC) [Nigeria] and ICF; 2019.

[35] Bhattacharya AA, Umar N, Audu A, Allen E, Schellenberg JRM, Marchant T (2019). Quality of routine facility data for monitoring priority maternal and newborn indicators in DHIS2: a case study from Gombe state, Nigeria. PLoS One.

[36] Gombe State Ministry of Health (SMoH). Gombe state framework for the implimentation of expanded access to family planning services [Internet]. Gombe (Nigeria); 2012. Available: https://www.fhi360.org/sites/default/files/media/documents/nigeria-gombe-state-framework.pdf.

[37] Tong A, Sainsbury P, Craig J (2007). Consolidated criteria for reporting qualitative research (COREQ): a 32-item checklist for interviews and focus groups. Int J Qual Heal Care.

[38] Boynton P, Greenhalgh T (2004). Selecting, designing, and developing your questionnaire. Bmj..

[39] Dworkin SL (2012). Sample size policy for qualitative studies using in-depth interviews. Arch Sex Behav.

[40] Bengtsson M (2016). How to plan and perform a qualitative study using content analysis. NursingPlus Open Elsevier.

[41] Guest G, Namey E, McKenna K (2017). How many focus groups are enough? Building an evidence base for nonprobability sample sizes. Field methods.

[42] Tynan AC, Drayton JL (1988). Conducting focus groups—a guide for first-time users. Mark Intell Plan.

[43] Ouédraogo Ali, Kiemtoré Sibraogo, Zamané Hyacinthe, Bonané Blandine T., Akotionga Michel, Lankoande Jean (2014). Respectful maternity care in three health facilities in Burkina Faso: The experience of the Society of Gynaecologists and Obstetricians of Burkina Faso. International Journal of Gynecology & Obstetrics.

[44] Bradley Susan, McCourt Christine, Rayment Juliet, Parmar Divya (2016). Disrespectful intrapartum care during facility-based delivery in sub-Saharan Africa: A qualitative systematic review and thematic synthesis of women's perceptions and experiences. Social Science & Medicine.

[45] Burrowes S, Holcombe SJ, Jara D, Carter D, Smith K. Midwives’ and patients’ perspectives on disrespect and abuse during labor and delivery care in Ethiopia: a qualitative study. BMC Pregnancy Childbirth. 2017;17:1–14. 10.1186/s12884-017-1442-1 PMC5567643 28830383

[46] Downe S, Lawrie TA, Finlayson K, Oladapo OT. Effectiveness of respectful care policies for women using routine intrapartum services: a systematic review. Reprod Health. 2018;15:1–13. 10.1186/s12978-018-0466-y PMC5801845 29409519

[47] Okafor Innocent I., Ugwu Emmanuel O., Obi Samuel N. (2014). Disrespect and abuse during facility-based childbirth in a low-income country. International Journal of Gynecology & Obstetrics.

[48] Igboanugo GM, Martin CH. What are pregnant women in a rural Niger Delta community’s perceptions of conventional maternity service provision? An exploratory qualitative study. [Internet]. African journal of reproductive health. Women’s Health and Action Research Centre; 2011. doi:10.2307/41762346 22574493

[49] Uzochukwu BSC, Onwujekwe OE, Akpala CO. Community satisfaction with the quality of maternal and child health services in Southeast Nigeria [Internet]. East Afr Med J. 2004. 10.4314/eamj.v81i6.9178. 10.4314/eamj.v81i6.9178 16167676

[50] Udoma EJ, Ekanem AD, Abasiattai AM, Bassey EA (2008). Reasons for preference of delivery in spiritual church-based clinics by women of south-South Nigeria. Niger J Clin Pract.

[51] Iyaniwura C, Yussuf Q (2009). Utilization of antenatal care and delivery services in Sagamu, south western Nigeria. Afr J Reprod Health.

[52] Al-Janabi Annegret, Al-Wahdani Batool, Ammar Walid, Arsenault Catherine, Asiedu Ernest Konadu, Etiebet Mary-Ann, Forde Ian, Gage Anna D, García-Saisó Sebastián, Guanais Frederico, Hansen Peter M, Hovig Dana, Jhalani Manoj, Kruk Margaret E, Maliqi Blerta, Marikar Kadar, Matsoso Malebona Precious, Pate Muhammad, Peterson Stefan, Roder-DeWan Sanam, Schulze Alexander, Somers Kate, Shiozaki Yasuhisa, Thapa Gagan (2018). Bellagio Declaration on high-quality health systems: from a quality moment to a quality movement. The Lancet Global Health.

[53] Heale R, Forbes D (2013). Understanding triangulation in research. Evid Based Nurs.

[54] Federal Ministry of Health (2013). Task Shifting and Task Sharing Policy for Maternal and Newborn Health.

[55] National Primary Health Care Development Agency. Community health influencers, promoters, and services Programme (CHIPS). Abuja; 2018. Available at https://nphcda.gov.ng/special-programmes/chips/. Accessed 10 Oct 2019

[56] Onah HE, Ikeako LC, Iloabachie GC (2006). Factors associated with the use of maternity services in Enugu, southeastern Nigeria. Soc Sci Med Pergamon.

[57] Moronkolaphd O A, Omonu J B, Iyayi D A, Tiamiyu M A (2007). Perceived determinants of the utilization of maternal health-care services by rural women in Kogi State, Nigeria. Tropical Doctor.

[58] Moore BM, Alex-Hart BA, George IO. Utilization of health care services by pregnant mothers during delivery: a community based study in Nigeria. East Afr J Public Health. 2011. 10.11694/pamj.supp.2014.17.1.3596. 22066284

[59] Ratcliffe HL, Sando D, Mwanyika-Sando M, Chalamilla G, Langer A, McDonald KP. Applying a participatory approach to the promotion of a culture of respect during childbirth. Reprod Health. 2016. 10.1186/s12978-016-0186-0. 10.1186/s12978-016-0186-0 PMC4948103 27424514

[60] Freedman Lynn P, Kruk Margaret E (2014). Disrespect and abuse of women in childbirth: challenging the global quality and accountability agendas. The Lancet.

[61] McMahon SA, Mnzava RJ, Tibaijuka G, Currie S (2018). The “hot potato” topic: challenges and facilitators to promoting respectful maternal care within a broader health intervention in Tanzania prof. Suellen Miller. Reprod Health.

[62] Oladapo OT, Bohren MA, Fawole B, Mugerwa K, Ojelade OA, Titiloye MA (2017). Negotiating quality standards for effective delivery of labor and childbirth care in Nigeria and Uganda. Int J Gynecol Obstet.

[63] Tourangeau R, Yan T (2007). Sensitive questions in surveys. Psychol Bull.

[64] Kruk ME, Kujawski S, Mbaruku G, Ramsey K, Moyo W, Freedman LP (2018). Disrespectful and abusive treatment during facility delivery in Tanzania: a facility and community survey. Health Policy Plan.

[65] Glick Peter (2009). How reliable are surveys of client satisfaction with healthcare services? Evidence from matched facility and household data in Madagascar. Social Science & Medicine.

[66] Sando David, Kendall Tamil, Lyatuu Goodluck, Ratcliffe Hannah, McDonald Kathleen, Mwanyika-Sando Mary, Emil Faida, Chalamilla Guerino, Langer Ana (2014). Disrespect and Abuse During Childbirth in Tanzania. JAIDS Journal of Acquired Immune Deficiency Syndromes.

