# Supplementary material for: Understanding mistreatment during institutional delivery in Northeast Nigeria: a mixed-method study
Source: Reprod Health. 2019 Dec 2;16:174. doi: 10.1186/s12978-019-0837-z (PMC6889445; doi:10.1186/s12978-019-0837-z)
Supplement: Supplementary file 1 — Additional file 1: Table S1. Mistreatment questions. [file 12978_2019_837_MOESM1_ESM.docx]

**Table S1. Mistreatment questions**

**During your most recent facility-based childbirth, did you experience any of the following (Yes/No)?**

| Beaten, pushed, pinched, slapped or poked by a birth attendant |
| --- |
| Physically restrained, tied or gagged by a birth attendant or other staff |
| Harsh or rude language, judgmental or accusatory comments toward you, by a birth attendant or other staff |
| Threats of withholding treatment or blamed for poor outcomes if you did not comply by a birth attendant or other staff |
| Treated negatively (act or gestures) because of your ethnicity/ race/ religion/ income/ HIV/ age by a birth attendant or other staff |
| Sexually abused, touched inappropriately, gestures suggestive of sexual interest, raped, by a birth attendant or other staff |
| Birth attendant performed medical examination/ virginal examination without explaining to you why and getting permission from you |
| Birth attendant or other staff discussed your confidential information about your health in a way that others could hear? |
| Birth attendant disregarded signs of pains displayed by you during vaginal examination. Denied you pain relief for any reason |
| Felt neglected, abandoned, long delays, or ignored by the birth attendant or staff |
| Birth attendant (doctor, nurse/midwife) absent at the time of delivery |
| Birth attendant performing surgical operations (e.g. Episiotomy) without providing you with any explanation about the procedure and getting permission from you or your family |
| Birth attendant did not encourage you to ask questions or respond to your questions, did not listen to you, did not give you any opportunity to discuss any preferences or requests |
| Health workers or staff dismissed your concerns (or companion), complains or fears |
| You were unable to get an interpreter when you needed an interpreter |
| Birth attendant or staff did not receive you warmly, did not show empathy, displayed non caring, nonchalant attitude toward you |
| Birth attendant or staff did not emotionally support you |
| Birth attendant disallowed you to have a birth companion present at any point |
| Birth attendant did not involve you in any decision making or informed you about decisions that they took regarding your care and the care of your baby |
| Birth attendant disallowed you to eat or drink or move around |
| Birth attendant disallowed you to deliver in the position you preferred |
| Birth attendant disallowed you to practice safe cultural practices e.g. not allowed to pray, have religious symbols, use of amulets |
| Not allowed to go home after delivery due to inability to pay hospital bills |
| Poor physical condition of facilities (crowded delivery room, dirty delivery room, not having a bed) |
| Felt there were no enough staff to look after you e.g. presence of at least one skilled birth attendant and at least one support staff during labour and delivery |
| Shortages of essential medicines and commodities e.g. you had to wait for your family had to go out and buy supplies before a service was provided regardless of the hour |
| Lack of curtains, partitions, or other measures used to provide privacy for you from others not involved in providing you care |
| Lack of mechanism that allows you or your companion to report a complain, displeasure or grievance with services received or denied when you needed to |
| Health worker requested monetary payment or non-monetary incentives from you or your family |
| Felt user fee structure was not clear e.g. health workers or staff did not provide you or your family information or access to price list for pharmaceutical products, hospital services related to labour and delivery |
| Felt request made by health workers was unreasonable e.g. asked to clean up your own blood, urine, faeces, fetch water, buy excess materials |
